# Nelaton’s Suppository

**Published:** 1876-05

**Authors:** 


"Nelaton's Suppository" is the best substitute for purgative medicines that we have ever seen; in fact, it is the only thing we know of that is a complete substitute for cathartics in most cases.

Before we had had much experience with it we prescribed only in cases of piles, and supposed it cured constipation by curing the piles; we have ascertained, however, that it almost always cures constipation, even where piles do not exist.

Many people write to us for "Nelaton's Suppository" and we send the orders to "Hall & Ruckel, Wholesale Druggist, 218 and 220 Greenwich Street, New York." It is better to send all orders for the "Suppository" directly to them.—[Ed. Hall's Journal of Health.]

